# Oral verruciform xanthoma: Report of 13 new cases and review of the literature

**DOI:** 10.4317/medoral.22342

**Published:** 2018-06-21

**Authors:** Paris Tamiolakis, Vasileios I. Theofilou, Konstantinos I. Tosios, Alexandra Sklavounou-Andrikopoulou

**Affiliations:** 1 DDS, Postgraduate Student, Department of Oral Medicine and Oral Pathology, School of Dentistry, National and Kapodistrian University of Athens, Greece, 2 Thivon Str, 115 27 Athens, Greece; 2 DDS, PhD, Assistant Professor, Department of Oral Medicine and Oral Pathology, School of Dentistry, National and Kapodistrian University of Athens, Greece, 2 Thivon Str, 115 27 Athens, Greece; 3 DDS, MSc, PhD, Professor, Head of Department of Oral Medicine and Oral Pathology, School of Dentistry, National and Kapodistrian University of Athens, Greece, 2 Thivon Str, 115 27 Athens, Greece

## Abstract

**Background:**

Oral verruciform xanthoma (OVX) is a rare lesion. The purpose of the present study is to describe the clinical features of 13 OVXs and review all cases reported in the English literature.

**Material and Methods:**

Thirteen cases of OVX diagnosed during a 47-year period were retrospectively collected. The patients’ gender and age, as well as the main clinical features of the lesions were retrieved from the biopsy request forms. Pubmed®, Scopus® and Google ScholarTM electronic databases were searched with the key word “verruciform xanthoma”. Only cases of histologically confirmed OVX were included in the study.

**Results:**

The 13 OVXs represented approximately 0.04% of 35,617 biopsies accessioned during the study period. They affected 13 patients, 8 males and 5 females with a mean age of 48.8±14 years. They mainly appeared as asymptomatic granular nodules or plaques, with elastic or normal consistency and white color, in the gingiva or hard palate. Literature review yielded 416 cases of OVX. With the addition of cases of the present study, 429 cases of OVX have been presented in the English literature. OVX has a slight male predominance with a male to female ratio of 1.4:1 and the majority of patients are in the 5th to 7th decade of life. Clinically, OVX mainly presents as an asymptomatic, single, papillary or granular plaque or nodule, with elastic or soft consistency and white, red or pink color. It measures approximately 1cm and is most commonly located on the gingiva, tongue, hard palate or buccal mucosa. The treatment of choice is surgical excision with little rates of recurrence.

**Conclusions:**

Verruciform xanthoma is a rare lesion most often encountered on the ginigival mucosa. As its clinical presentation is not pathognomonic, it should be included in the differential diagnosis of verrucous or papillary lesions.

** Key words:**Oral verruciform xanthoma, oral tumors.

## Introduction

Verrucifrom xanthoma is a rare benign lesion of unknown pathogenesis, first described in the oral cavity by Shafer in 1971 (1). Similar lesions have been reported in the anogenital area (2), i.e. the penis, the scrotum and vulva (3), as well as in the skin, the latter usually in association with chronic inflammatory skin diseases such as dystrophic epidermolysis bullosa and cutaneous discoid lupus erythematous, chronic or congenital lymphoedema, graft versus host disease, and congenital epidermal nevi (2).

Oral verruciform xanthoma (OVX) manifests as an asymptomatic, solitary, sharply demarcated and slightly raised plaque usually located on the gingiva, with a papillary, granular or verrucous surface, and red or pink color (4). It shows a slight male predilection and most patients are in the 5th to 7th decade of life (4). Microscopic examination discloses a papillary or verrucous proliferation of hyperparakeratotic squamous epithelium with a characteristic orange hue, and aggregation of “foamy or xanthoma cells” within the connective tissue (4-6). The ret pegs are elongated with uniform depth (4,7) and deep, keratin-filled clefts may be noticed between the epithelial projections (4). Exocytosis of neutrophils in the parakeratin layer and infiltration of lymphocytes and plasma cells in the connective tissue may also be seen (7). The xanthoma cells are macrophages of varying size with eccentrically placed nuclei (4,8); they are mostly confined to the connective tissue papillae, but may extend beyond the level of the rete ridges (7,8). They may replace all connective tissue elements in the papillae or be sparsely present (4,7).

We report the clinical features of 13 new cases of OVX and review the pertinent English literature with regard to the clinical and demographic features, etiology, pathogenesis and treatment.

## Material and Methods

The study was approved by the Research Ethics Committee of Athens Dental School (code number 349/08.11.2017).

Fourteen cases of OVX were retrospectively retrieved from the files of the Department of Oral Medicine and Pathology from January 1971 to July 2017. The diagnosis in each case was reconfirmed by studying representative hematoxylin and eosin stained tissue sections. The age and gender of the patients, as well as location and main clinical features of the lesions were collected from the biopsy request forms and tabulated.

For the literature review, Pubmed®, Scopus® and Google ScholarTM electronic databases were searched using the keyword “verruciform xanthoma” up to July 15, 2017. All cases of microscopically confirmed VX located in the oral mucosa that were published in the English literature were retrieved. The references of all retrieved publications were manually searched for additional cases. Multiple lesions or recurrences were considered as OVX only when they were microscopically confirmed. The age and gender of the patients, location and main clinical features of the lesions, as well as follow-up data were collected and tabulated.

## Results

Out of 35,617 biopsies accessioned during the study period, 14 cases of solitary OVX were found, representing 0.04% of total number. As one of those cases had been previously described in the English literature (9), the clinical features of the 13 new cases are tabulated in  Table 1. Eight patients (61.5%) were males and 5 (38.5%) were females, the male to female ratio being 1.6:1. Age was reported in eleven patients and ranged from 28 to 73 years (mean age 48.8±14 years, median age 48 years). The mean age of the males (mean age 42.1±11.6 years, median 38 years) was younger than that of the females (mean age 60.5±9.4 years, median 60.5 years), the difference being statistically significant (*p*<0.05). Most lesions were clinically described as asymptomatic nodules (6 cases, 46.2%), plaques (5 cases, 38.5%) or ulcers (2 cases, 15.4%), of white (8 cases, 72.7%), pink (2 cases, 18.2%), or red (1 case, 9.1%) color. Their surface was granular (6 cases, 54.5%), rough (2 cases, 18.2%), papillary (2 cases, 18.2%) or verrucous (1 case, 9.1%). Consistency was described as elastic (5 cases, 62.5%), normal (2 cases, 25%) or soft (1 case, 12.5%). Size was reported in 9 cases and approximately half of them measured less than 1cm in largest dimension (range 0.3-2.5cm, mean 1±0.7cm, median 0.7cm). Most lesions were located on the gingiva (5 cases, 38.5%), followed by the hard palate (3 cases, 23.1%), lateral border of the tongue (2 cases, 15.4%), lower lip (2 cases, 15.4%) and alveolar mucosa (1 case, 7.7%).

**Figure 1:**
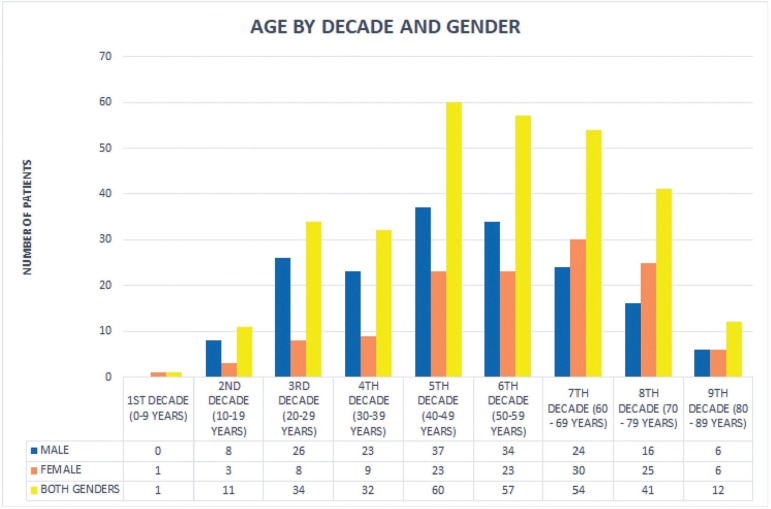
Age of patients related to gender and decade of life.

The literature review yielded 74 publication with a total number of 416 cases of OVX (1,4,9-73). With the addition of the 13 new cases of the present study, the total number of OVXs reported in the English literature is 429. There was a slight male predominance with 233 cases (58%) occurring in male patients and 169 cases (42%) in females (male to female ratio 1.4:1). Age ranged from 2.5 to 89 years, with the majority of patients being in the 5th to 7th decade of life (mean age 51 years). The mean age of males (47.6 years) was younger than that of females (55.8 years), the difference being statistically significant (*p*<0.05). Male to female ratio before the age of 50 years was 2.1:1, but after the age of 50 years is approximately 1:1. Age distribution according to gender is shown in Fig 1. OVX usually manifested as a single lesion (341 cases, 98.6%), but in 5 cases (4,24,42,55,71) multiple lesions were seen. There were, also, 2 cases with multiple lesions (39,72), but biopsy was performed in only one of them. In one case (55) 4 OVXs were present and in another 4 cases 2 lesions were detected (4,24,42,71). In 2 cases, OVX manifested concurrently with extraoral lesions (25,66), while one case with multifocal lesions was described as “disseminated” verrucifom xanthoma (74). The majority of OVXs were located on the gingiva (182 cases, 45.3%), followed by the tongue (53 cases, 13.2%), hard palate (51 cases, 12.7%), buccal mucosa (37 cases, 9.2%), alveolar mucosa (25 cases, 6.2%), floor of the mouth (15 cases, 3.7%), lips (15 cases, 3.7%), soft palate (9 cases, 2.2%), maxillary buccal vestibule (4 cases, 1%), vermillion border of the lower lip (3 cases, 0.7%), palatal junction (3 cases, 0.7%) and lower mucobuccal fold (2 cases, 0.5%). Single cases were also seen on the retromolar pad (0.3%), mandibular posterior gingiva with extension to the buccal mucosa (0.3%) and maxillary anterior gingiva extending to the upper lip (0.3%). Clinically, in the majority of cases, OVX presented as a plaque (19 cases, 38%), nodule (11 cases, 22%) or mass (6 cases, 12%), of white (22 cases, 29%), red (15 cases, 19.7%), pink (14 cases, 18.4%) or yellow (10 cases, 13.2%) color, with elastic (6 cases, 40%) or soft (6 cases, 40%), consistency. The surface was papillary (35 cases, 38.5%), granular (22 cases, 24.2%), or verrucous (17 cases, 18.7%). Most lesions measured approximately 1cm in major diameter (mean size 1±0.7cm, median size 1cm). In 95.7% of the cases no symptoms were present, justifying the long duration of 12.8±19.7 months (range 2 weeks-12 years, median duration 6 months) before diagnosis. In one case (11) a barely discernible radiolucency could be noticed upon radiographic examination. The follow up period ranged from 2 months – 18 years (mean follow-up time 8.9±8 years, median follow-up time 3 years) and 6 cases recurred (12%). In 99.1% of the cases, surgical excision was performed, whereas in 2 cases diagnosis was established after incisional biopsy followed by total excision with electro-cautery knife (28) or carbon dioxide laser (49). In one case (66) imiquimod was locally applied after surgical excision.

**Table 1:**
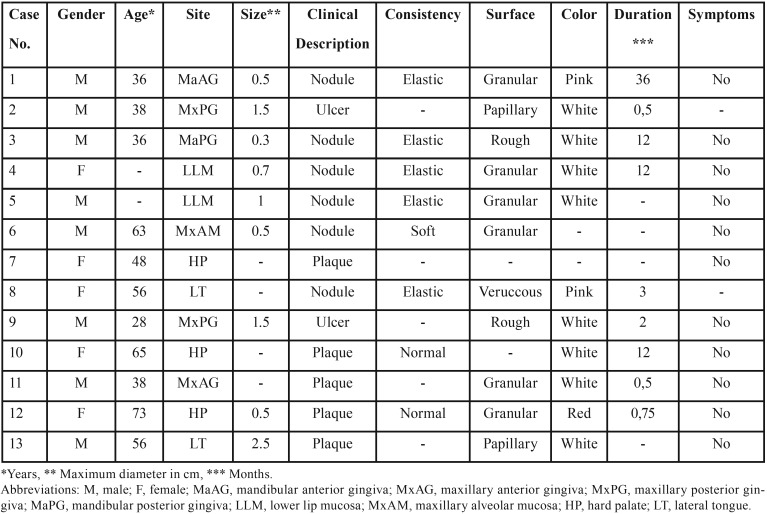
Demographics and clinical characteristics of 13 cases of oral verruciform xanthoma.

Cases of OVX was described in patients with oral lichen planus (10 cases) (7,15,16,32,35,41,48,65); graft versus host disease (7 cases) (4,29,46); bone marrow transplantation (5 cases) (49,53,66, cases 4 and 5 of the present study); oral squamous cell carcinoma (4 cases) (15,26,41,65); leukoplakia (1 case) (7); amyloidosis (1 case) (7); in patients under chemotherapy and radiotherapy for lymphoma (2 cases) (8); oral pemphigus vulgaris (1 case) (19), oral discoid lupus erythematous (1 case) (37), submucosal fibrosis (1 case) (41), neurofibromatosis (1 case) (47) and paracoccioidiomycosis (1 case) (65). In three cases, OVX occurred simultaneously with osteoma (8), carcinoma in situ (26) and warthy dyskeratoma (30).

## Discussion

OVX is a rare lesion, as Shafer (1) reported 15 cases (0,045%) among 33,000 biopsies during a 23 year period and Jones and Franklin (75) 9 cases (0.02%) among 44,007 biopsies during a 30 year period. The 14 cases retrieved from the files of our Department represent 0.04% of 35.617 biopsies diagnosed during a 47 year period. In their review Philipsen *et al.* (4) found 199 cases of OVX in the English literature and 83 cases in the Japanese literature. Since this review 134 cases have been published in the English literature that in addition to the 13 new cases found in our files raise the total number of OVX to 429 cases. The more common occurrence of OVX in males than females and in middle-aged patients (4) was confirmed in the present study. The clear male predominance of the lesion in patients younger than 50 years of age, as well as their tendency to occur almost a decade earlier in males compared to females cannot be explained. The gingiva is the most commonly involved oral site, followed by the tongue, hard palate, and buccal mucosa (4).

The clinical differential diagnosis includes other verrucous lesions, such as verrucous leukoplakia, verrucous carcinoma, squamous papilloma, verruca vulgaris, condyloma acuminatum and squamous cell carcinoma (46,63).

Diagnosis requires microscopic examination. Nowparast *et al.* (17) have described three histologic subtypes of OVX that do not correlate with clinical or prognostic parameters, and most probably represent various stages of the disease progression (25).

Immunohistochemically, parakeratin is negative for cytokeratin (CK) 14, in contrast to normal oral epithelium and Syndecan 1/CD138 shows granular positivity compared to normal oral mucosa, where a membranous pattern is seen (65). The foam cells are positive for CD68 and negative for S-100, positive for PAS and PAS-diastase (6,8) and some of them may represent transformed fibroblasts (7).

The pathogenesis of OVX is unknown. Unlike skin xanthomas no disturbance of lipid metabolism is reported, with the exception of one case (25) in a girl with an undefined systemic lipid disorder who developed a verruciform xanthoma on the tongue and in other parts of her body. A missense mutation in exon 6 of the 3β-hydroxysteroid dehydrogenase (NSDHL) gene that plays an important role in cholesterol biosynthesis was found in 22% of cutaneous xanthomas, but this mutation has not been examined in OVX (76). As in cutaneous lesions (2), OVX is not related to HPV (25,33,38). Occurrence of most OVX on the masticatory mucosa lead to the hypothesis that a “local irritant”, such as trauma or inflammation, may cause epithelial degeneration and that the degenerated epithelium forms lipids scavenged by macrophages (12,13). However, this theory does not explain the occurrence of OVX in sites where trauma is not common, such as the soft palate or floor of the mouth (4), the lack of degenerative epithelial cells on microscopic examination (8,9) and the persistence of foamy cells in the connective tissue (5). Some authors consider the epithelial changes as secondary to the presence of foamy cells (17,25) or “illusionary” (8) due to the upward pushing effect from the macrophages.

Accumulation of T lymphocytes, decreased number of Langerhans cells (8) and active antigen presentation in the epithelium of OVX, especially at the site of intense inflammation (33), suggest a local immunologic reaction (5). Accumulation of an inflammatory infiltrate predominated by T lymphocytes to an unknown antigen may act as a chemoattractant to macrophages that through the expression of epithelial adhesion molecules persist in the lesion (5). According to Ide *et al.* (7), periodontopathic pathogens, tobacco, mechanical stimuli, alcohol, foodstuff sensitizing or allergic agents or dental materials and drugs, alter keratinocytes causing chronic epithelial damage and trigger inflammation, with the predominance of T lymphocytes. T lymphocytes in turn stimulate keratinocytes to release cytokines and chemokines, thus attracting neutrophils (by IL-8 of the upper spinous keratinocytes) and additional T cells thus perpetuating T cell infiltration. T lymphocytes activate MCP-1 in the basal layer cells, which is a monocyte/macrophage cells attractor, thus attracting macrophages that express CCR2, the receptor of MCP-1. Macrophages trap and internalize lipids released by degenerative keratinocytes, thus becoming foamy. Consequently, macrophages oxidize lipids causing self induced-toxicity and eventually necrosis. The inflammatory response to necrosis attracts new macrophages, thus perpetuating their presence in OVX (7). Finally Shahrabi Farahani *et al.* (46) proposed that disorders which lead to basal cells damage such as lichen planus, GVHD, pemphigus vulgaris and epidermolysis bullosa may also trigger the formation of OVX.

OVX has good prognosis. Only 6 cases with recurrence after surgical excision (12%) (mean time of recurrence 2.8±1.5 years) were recorded (17,22,33,7,47,57). This underlines the necessity for a follow-up. No case of malignant transformation has been reported. In one case squamous cell carcinoma developed in the area where an OVX was present four years earlier, but this was considered coincidental (6).

## Conclusions

Verruciform xanthoma is a rare lesion most often encountered on the ginigival mucosa. As its clinical presentation is not pathognomonic, it should be included in the differential diagnosis of verrucous or papillary lesions.
